# Supplementary material for: Opioid analgesia alters corticospinal coupling along the descending pain system in healthy participants
Source: eLife. 2022 Apr 26;11:e74293. doi: 10.7554/eLife.74293 (PMC9042228; doi:10.7554/eLife.74293)
Supplement: Supplementary file 1. [file elife-74293-supp1.docx]

**Supplementary file 1a**

| Stimulus type | difference of VAS means | t | p_cuncorr_ | p_corr_ |
| --- | --- | --- | --- | --- |
|  |  |  |  |  |
| **NaCl vs. Remi (df = 76)** | | | | |
| Pooled | 15.75 | 5.56 | < 0.001 | < 0.001 |
| Constant T1 | 15.07 | 4.38 | < 0.001 | < 0.001 |
| Constant T2 | 17.14 | 4.89 | < 0.001 | < 0.001 |
| Stepwise T1 | 18.45 | 6.31 | < 0.001 | < 0.001 |
| Stepwise T2 | 12.35 | 3.77 | < 0.001 | 0.003 |
| Constant online | 12.74 | 3.94 | < 0.001 | 0.002 |
| Stepwise online | 12.79 | 5.75 | < 0.001 | < 0.001 |
| Mood rating | 0.29 | 2.15 | 0.035 | - |
|  |  |  |  |  |
| **Remi50 vs. Remi100 (df = 51)** | | | |  |
| Pooled | 0.18 | 0.06 | 0.95 | 1 |
| Constant T1 | 3.1 | 0.78 | 0.44 | 1 |
| Constant T2 | 1.25 | 0.33 | 0.74 | 1 |
| Stepwise T1 | -0.06 | 0.02 | 0.99 | 1 |
| Stepwise T2 | -3.57 | 1.07 | 0.29 | 1 |
| Constant online | 3.65 | 1.02 | 0.31 | 1 |
| Stepwise online | 1.89 | 0.72 | 0.47 | 1 |
| Mood rating | 0.01 | 0.07 | 0.94 | - |

**Supplementary file 1b**

| x | y | z | voxels | t | z | p_corr_ |
| --- | --- | --- | --- | --- | --- | --- |
| **Interaction Remi > NaCl (brain)** | | | | | | |
| 56 | 9 | 3 | 153 | 5.95 | 5.38 | < 0.001 |
| 33 | 10 | 4 | 35 | 5.80 | 5.26 | 0.001 |
| -34 | 16 | 3 | 82 | 5.13 | 4.74 | 0.008 |
| -38 | 12 | 8 |  | 5.05 | 4.68 | 0.011 |
| -33 | 15 | 12 |  | 4.67 | 4.37 | 0.037 |
| 62 | -24 | 24 | 14 | 4.94 | 4.59 | 0.015 |
| 6 | -21 | 10 | 4 | 4.71 | 4.40 | 0.033 |
| -42 | 12 | -2 | 3 | 4.66 | 4.35 | 0.039 |
|  |  |  |  |  |  |  |
| **Interaction Remi > NaCl (spinal)** | | | | | | |
| -5 | -47 | -151 | 26 | 4.24 | 4.01 | 0.013 |
| 0 | -45 | -150 | 59 | 4.17 | 3.94 | 0.016 |
| -3 | -45 | -147 | 37 | 3.97 | 3.78 | 0.028 |
|  |  |  |  |  |  |  |
| **Interaction NaCl > Remi (brain)** | | | | | | |
| 0 | 20 | -9 | 40 | 4.98 | 4.61 | 0.004 |
|  | | | | | | |

**Supplementary file 1c**

| x | y | z | voxels | t | z | p_corr_ |
| --- | --- | --- | --- | --- | --- | --- |
| **Negative correlation** | | | | | | |
| 57 | 6 | 3 | 111 | 6.95 | 6.09 | < 0.001 |
| 50 | -28 | 24 | 325 | 6.31 | 5.64 | < 0.001 |
| 64 | -33 | 32 |  | 5.93 | 5.36 | < 0.001 |
| 60 | -24 | 26 |  | 5.38 | 4.93 | 0.004 |
| 52 | -36 | 32 |  | 4.96 | 4.61 | 0.015 |
| 16 | -14 | 9 | 96 | 6.20 | 5.56 | < 0.001 |
| -42 | 0 | 4 | 41 | 5.36 | 4.92 | 0.004 |
| 4 | -18 | 12 | 13 | 5.19 | 4.79 | 0.007 |
| 9 | -22 | 12 |  | 4.85 | 4.51 | 0.021 |
| -34 | 9 | 14 | 17 | 5.18 | 4.78 | 0.008 |
| -10 | -15 | 0 | 12 | 5.07 | 4.69 | 0.010 |
| 54 | -16 | 20 | 11 | 4.94 | 4.58 | 0.016 |
| 42 | -2 | -8 | 3 | 4.79 | 4.46 | 0.026 |
| -8 | -33 | -8 | 3 | 4.64 | 4.34 | 0.042 |
| -8 | -16 | -6 | 1 | 4.63 | 4.33 | 0.043 |
|  |  |  |  |  |  |  |
|  |  |  |  |  |  |  |
| **Positive correlation** | | | | | | |
| 8 | 24 | -20 | 125 | 5.05 | 4.67 | 0.003 |
| 6 | 51 | -6 | 91 | 5.03 | 4.65 | 0.003 |
| 3 | 63 | -15 | 2 | 4.93 | 4.58 | 0.005 |
| -6 | 54 | -4 | 5 | 4.26 | 4.02 | 0.039 |
|  |  |  |  |  |  |  |

**Supplementary file 1d**

| x | y | z |  | t | z | p_corr_ |
| --- | --- | --- | --- | --- | --- | --- |
| **Interaction Remi > NaCl (brain)** | | | | | | |
| 33 | 10 | 6 |  | 5.41 | 4.96 | 0.014 |
| 58 | -24 | 24 |  | 4.67 | 4.36 | 0.04 |
|  |  |  |  |  |  |  |
| **Interaction Remi > NaCl (spinal)** | | | | | | |
| -3 | -45 | -147 |  | 4.41 | 4.15 | 0.008 |
| 0 | -46 | -152 |  | 4.18 | 3.95 | 0.015 |
|  |  |  |  |  |  |  |
| **Interaction NaCl > Remi (brain)** | | | | | | |
| 3 | 50 | -6 |  | 4.30 | 4.05 | 0.036 |
| 3 | 18 | -16 |  | 4.23 | 4.00 | 0.044 |
|  |  |  |  |  |  |  |
| **Negative correlation** | | | | | | |
| 32 | 15 | 9 |  | 5.46 | 5.00 | 0.003 |
| -42 | 2 | 6 |  | 4.87 | 4.53 | 0.02 |
| 50 | -30 | 24 |  | 4.83 | 4.50 | 0.023 |
| 60 | -26 | 27 |  | 4.67 | 4.36 | 0.039 |
|  |  |  |  |  |  |  |
| **Positive correlation** | | | | | | |
| 6 | 52 | -6 |  | 4.83 | 4.50 | 0.007 |
| 6 | 58 | -16 |  | 4.47 | 4.20 | 0.021 |
|  |  |  |  |  |  |  |

**Supplementary file 1e**

| x | y | z | voxels | t | z | p_corr_ |
| --- | --- | --- | --- | --- | --- | --- |
|  |  |  |  |  |  |  |
| **PPI DH -> PAG** | | | | | | |
| -8 | -26 | -6 | 72 | 4.93 | 4.57 | < 0.001 |
| -4 | -30 | -8 |  | 4.44 | 4.16 | 0.002 |
|  |  |  |  |  |  |  |
| **PPI vmPFC -> PAG** | | | | | | |
| 8 | -33 | -8 | 101 | 5.73 | 5.19 | < 0.001 |
| -6 | -24 | -4 | 4 | 3.53 | 3.38 | 0.026 |
|  |  |  |  |  |  |  |
| **PPI PAG -> DH** | | | | | | |
| -3 | -47 | -150 | 64 | 3.89 | 3.71 | 0.037 |

**Supplementary file 1f**

|  | NaCl | Remi50 | Remi100 |
| --- | --- | --- | --- |
| N | 25 | 26 | 27 |
| Age (years) | 24.48 (3.72) | 25.88 (3.29) | 25.74 (4.22) |
| Handedness (r/l) | 23/2 | 21/5 | 21/6 |
| Pain threshold (°C) | 42.92 (2.37) | 43.06 (2.31) | 43.32 (2.45) |
| Temp 50 VAS (°C) | 43.76 (1.04) | 44.10 (0.75) | 44.44 (0.67) |
| Weight (kg) | 86.01 (12.07) | 80.15 (8.70) | 78.27 (10.78) |
| Height (cm) | -^#^ | 182.23 (5.56)^+^ | 179.72 (6.71) |
| BMI | -^#^ | 23.92 (2.51) ^+^ | 24.26 (3.19) |
| Body fat (%) | -^#^ | 16.7 (5.39) ^+^ | 17.47 (6.36) |
| Remi rate (ml/h) | 0 | 23.98 (2.58) | 23.41 (3.25) |
| Belief remi (y/n) | 9/14* | 22/3* | 26/0* |
| ΔHeart rate° | -1.55 (2.54) | -0.45 (6.27) | 1.01 (5.00) |
| ΔRespiration rate° | 0.41 (1.35) | 1.82 (3.55) | 0.55 (2.70) |
|  |  |  |  |
| BDI | 1.88 (2.49) | 2.27 (2.26) | 1.67 (2.13) |
| STAI-T | 29.12 (4.99) | 30.89 (7.30) | 32.26 (7.76) |
| STAI-S | 30 (5.92) | 31.42 (7.12) | 31.11 (6.66) |
| SDS | 14.4 (3.57) | 12.96 (3.98) | 13.30 (4.85) |
| BMQ | 13.73 (6.31) | 13.75 (5.67) | 13.32 (5.52) |
|  |  |  |  |
| UAW pre | 3.36 (3.13) | 2.89 (2.3) | 4.27 (3.12) |
| UAW post | 3.56 (4.3) | 4.31 (4.86) | 4.44 (4.51) |
| MDMQ G-B pre | 17.88 (1.94) | 17.69 (2.02) | 17.26 (2.25) |
| MDMQ G-B post | 17.46 (2.69) | 17.04 (2.84) | 17.89 (1.83) |
| MDMQ A-T pre | 16.5 (2.55) | 16.73 (2.29) | 15.93 (2.62) |
| MDMQ A-T post | 14.58 (3.48) | 14.15 (3.52) | 13.48 (3.24) |
| MDMQ C-N pre | 16.5 (2.47) | 16.89 (2.46) | 16.63 (2.65) |
| MDMQ C-N post | 17.67 (2.12) | 16.96 (2.44) | 17.67 (2.66) |

^# Data were not acquired in participants of the NaCl group^

^+ Missing data in 2 subjects^

^* Missing data in 4 subjects (2/1/1 respectively per group)^

^° Baseline – treatment phase^

**Supplementary file 1g**

| **Cases** | **Sum of Squares** | **df** | **Mean Square** | **F** | **p** |
| --- | --- | --- | --- | --- | --- |
| Group | 4811.882 | 2 | 2405.941 | 2.636 | 0.078 |
| Time | 20461.791 | 1 | 20461.791 | 73.98 | < .001 |
| Time ✻ Group | 8448.324 | 2 | 4224.162 | 15.273 | < .001 |
| RatingType | 109403.216 | 3 | 36467.739 | 259.096 | < .001 |
| RatingType ✻ Group | 390.041 | 6 | 65.007 | 0.462 | 0.836 |
| Time ✻ RatingType | 412.362 | 3 | 137.454 | 4.313 | 0.006 |
| Time ✻ RatingType ✻ Group | 328.171 | 6 | 54.695 | 1.716 | 0.118 |
|  |  |  |  |  |  |
|  |  | **Mean Difference** | **SE** | **t** | **p_holm_** |
| **Post Hoc Comparisons - Time** | |  |  |  |  |
| Baseline | Treatment | 11.458 | 1.332 | 8.601 | < .001 |
|  |  |  |  |  |  |
| **Post Hoc Comparisons - Group ✻ Time** | |  |  |  |  |
| NaCl, Baseline | Remi100, Baseline | -1.097 | 3.384 | -0.324 | 1 |
|  | Remi50, Baseline | -3.623 | 3.416 | -1.061 | 1 |
|  | NaCl, Treatment | 0.945 | 2.352 | 0.402 | 1 |
|  | Remi100, Treatment | 15.528 | 3.384 | 4.588 | < .001 |
|  | Remi50, Treatment | 13.182 | 3.416 | 3.859 | 0.002 |
| Remi100, Baseline | Remi50, Baseline | -2.527 | 3.35 | -0.754 | 1 |
|  | NaCl, Treatment | 2.042 | 3.384 | 0.603 | 1 |
|  | Remi100, Treatment | 16.625 | 2.263 | 7.346 | < .001 |
|  | Remi50, Treatment | 14.279 | 3.35 | 4.262 | < .001 |
| Remi50, Baseline | NaCl, Treatment | 4.568 | 3.416 | 1.338 | 1 |
|  | Remi100, Treatment | 19.152 | 3.35 | 5.716 | < .001 |
|  | Remi50, Treatment | 16.805 | 2.306 | 7.287 | < .001 |
| NaCl, Treatment | Remi100, Treatment | 14.583 | 3.384 | 4.309 | < .001 |
|  | Remi50, Treatment | 12.237 | 3.416 | 3.583 | 0.004 |
| Remi100, Treatment | Remi50, Treatment | -2.346 | 3.35 | -0.7 | 1 |
|  |  |  |  |  |  |
| **Post Hoc Comparisons - RatingType** | |  |  |  |  |
| Control1 | Control2 | 6.756 | 1.344 | 5.027 | < .001 |
|  | Stepwise1 | -24.258 | 1.344 | -18.05 | < .001 |
|  | Stepwise2 | 9.221 | 1.344 | 6.861 | < .001 |
| Control2 | Stepwise1 | -31.015 | 1.344 | -23.077 | < .001 |
|  | Stepwise2 | 2.465 | 1.344 | 1.834 | 0.068 |
| Stepwise1 | Stepwise2 | 33.479 | 1.344 | 24.911 | < .001 |
|  |  |  |  |  |  |
| **Post Hoc Comparisons - RatingType** | |  |  |  |  |
| Control1 | Control2 | 6.756 | 1.344 | 5.027 | < .001 |
|  | Stepwise1 | -24.258 | 1.344 | -18.05 | < .001 |
|  | Stepwise2 | 9.221 | 1.344 | 6.861 | < .001 |
| Control2 | Stepwise1 | -31.015 | 1.344 | -23.077 | < .001 |
|  | Stepwise2 | 2.465 | 1.344 | 1.834 | 0.068 |
| Stepwise1 | Stepwise2 | 33.479 | 1.344 | 24.911 | < .001 |
|  |  |  |  |  |  |
|  |  |  |  |  |  |
| **Post Hoc Comparisons - Time ✻ RatingType** | |  |  |  |  |
| Baseline, Control1 | Treatment, Control1 | 9.037 | 1.545 | 5.848 | < .001 |
|  | Baseline, Control2 | 4.575 | 1.488 | 3.074 | 0.011 |
|  | Treatment, Control2 | 17.975 | 1.946 | 9.239 | < .001 |
|  | Baseline, Stepwise1 | -25.912 | 1.488 | -17.409 | < .001 |
|  | Treatment, Stepwise1 | -13.567 | 1.946 | -6.973 | < .001 |
|  | Baseline, Stepwise2 | 8.214 | 1.488 | 5.519 | < .001 |
|  | Treatment, Stepwise2 | 19.266 | 1.946 | 9.902 | < .001 |
| Treatment, Control1 | Baseline, Control2 | -4.462 | 1.946 | -2.293 | 0.068 |
|  | Treatment, Control2 | 8.938 | 1.488 | 6.005 | < .001 |
|  | Baseline, Stepwise1 | -34.949 | 1.946 | -17.963 | < .001 |
|  | Treatment, Stepwise1 | -22.604 | 1.488 | -15.187 | < .001 |
|  | Baseline, Stepwise2 | -0.823 | 1.946 | -0.423 | 0.773 |
|  | Treatment, Stepwise2 | 10.229 | 1.488 | 6.872 | < .001 |
| Baseline, Control2 | Treatment, Control2 | 13.4 | 1.545 | 8.671 | < .001 |
|  | Baseline, Stepwise1 | -30.487 | 1.488 | -20.483 | < .001 |
|  | Treatment, Stepwise1 | -18.142 | 1.946 | -9.325 | < .001 |
|  | Baseline, Stepwise2 | 3.639 | 1.488 | 2.445 | 0.06 |
|  | Treatment, Stepwise2 | 14.691 | 1.946 | 7.551 | < .001 |
| Treatment, Control2 | Baseline, Stepwise1 | -43.887 | 1.946 | -22.557 | < .001 |
|  | Treatment, Stepwise1 | -31.542 | 1.488 | -21.192 | < .001 |
|  | Baseline, Stepwise2 | -9.761 | 1.946 | -5.017 | < .001 |
|  | Treatment, Stepwise2 | 1.291 | 1.488 | 0.867 | 0.773 |
| Baseline, Stepwise1 | Treatment, Stepwise1 | 12.345 | 1.545 | 7.988 | < .001 |
|  | Baseline, Stepwise2 | 34.126 | 1.488 | 22.928 | < .001 |
|  | Treatment, Stepwise2 | 45.178 | 1.946 | 23.22 | < .001 |
| Treatment, Stepwise1 | Baseline, Stepwise2 | 21.781 | 1.946 | 11.195 | < .001 |
|  | Treatment, Stepwise2 | 32.833 | 1.488 | 22.06 | < .001 |
| Baseline, Stepwise2 | Treatment, Stepwise2 | 11.052 | 1.545 | 7.151 | < .001 |

**Supplementary file 1h**

| x | y | z | voxels | t | z | P_FWE_ |
| --- | --- | --- | --- | --- | --- | --- |
|  | | | | | | |
| **Interaction Remi > NaCl (brain)** | | | | | | |
| 56 | 9 | 3 | 45 | 5.95 | 5.38 | 0.004 |
| 33 | 10 | 4 | 4 | 5.80 | 5.26 | 0.007 |
|  |  |  |  |  |  |  |
| **Interaction NaCl > Remi (brain)** | | | | | | |
| -62 | -21 | -12 | 7 | 5.56 | 5.07 | 0.017 |
| -20 | -38 | -15 | 2 | 5.42 | 4.97 | 0.028 |
| 52 | -25 | -27 | 1 | 5.37 | 4.93 | 0.033 |
|  |  |  |  |  |  |  |
| **Negative correlation** | | | | | | |
| 57 | 6 | 3 | 55 | 6.95 | 6.09 | < 0.001 |
| -4 | -21 | 14 | 55 | 6.92 | 6.07 | < 0.001 |
| 4 | -18 | 14 |  | 5.71 | 5.19 | 0.010 |
| 50 | -28 | 24 | 58 | 6.31 | 5.74 | 0.001 |
| 16 | -14 | 9 | 48 | 6.20 | 5.56 | 0.002 |
| 64 | -33 | 32 | 20 | 5.93 | 5.36 | 0.005 |
| 60 | -24 | 26 | 2 | 5.38 | 4.93 | 0.033 |
| -42 | 0 | 4 | 2 | 5.36 | 5.92 | 0.035 |
| 18 | -78 | -32 | 3 | 5.33 | 4.90 | 0.038 |
|  |  |  |  |  |  |  |
| **Positive correlation** | | | | | | |
| -64 | -21 | -14 | 3 | 5.41 | 4.96 | 0.030 |
| 34 | 36 | -2 | 2 | 5.36 | 4.92 | 0.036 |
|  |  |  |  |  |  |  |
| **PPI DH -> PAG** | | | | | | |
| -27 | -18 | -10 | 10 | 5.74 | 5.20 | 0.011 |
| -9 | -24 | -6 | 2 | 5.46 | 4.99 | 0.030 |
|  |  |  |  |  |  |  |
| **PPI vmPFC -> PAG** | | | | | | |
| 8 | -33 | -8 | 8 | 5.73 | 5.19 | 0.010 |
| -22 | -57 | 6 | 2 | 5.32 | 4.88 | 0.042 |
|  |  |  |  |  |  |  |
